# Host alarm calls attract the unwanted attention of the brood parasitic common cuckoo

**DOI:** 10.1038/s41598-019-54909-1

**Published:** 2019-12-06

**Authors:** Attila Marton, Attila Fülöp, Katalin Ozogány, Csaba Moskát, Miklós Bán

**Affiliations:** 1 Department of Evolutionary Zoology and Human Biology, University of Debrecen, Debrecen, Hungary; 2 Juhász-Nagy Pál Doctoral School, University of Debrecen, Debrecen, Hungary; 3 MTA-DE Behavioural Ecology Research Group, Department of Evolutionary Zoology and Human Biology, University of Debrecen, Debrecen, Hungary; 4 MTA-ELTE-MTM Ecology Research Group, a joint research group of the Hungarian Academy of Sciences, The Biological Institute of the Eötvös Loránd University and the Hungarian Natural History Museum, Budapest, Hungary

**Keywords:** Behavioural ecology, Evolutionary ecology

## Abstract

It is well known that avian brood parasites lay their eggs in the nests of other bird species, called hosts. It remains less clear, however, just how parasites are able to recognize their hosts and identify the exact location of the appropriate nests to lay their eggs in. While previous studies attributed high importance to visual signals in finding the hosts’ nests (e.g. nest building activity or the distance and direct sight of the nest from vantage points used by the brood parasites), the role of host acoustic signals during the nest searching stage has been largely neglected. We present experimental evidence that both female and male common cuckoos *Cuculus canorus* pay attention to their host’s, the great reed warbler’s *Acrocephalus arundinaceus* alarm calls, relative to the calls of an unparasitized species used as controls. Parallel to this, we found no difference between the visibility of parasitized and unparasitized nests during drone flights, but great reed warblers that alarmed more frequently experienced higher rates of parasitism. We conclude that alarm calls might be advantageous for the hosts when used against enemies or for alerting conspecifics, but can act in a detrimental manner by providing important nest location cues for eavesdropping brood parasites. Our results suggest that host alarm calls may constitute a suitable trait on which cuckoo nestlings can imprint on to recognize their primary host species later in life. Our study contributes to the growing body of knowledge regarding the context-dependency of animal signals, by providing a novel example of a beneficial acoustic trait intercepted by a heterospecific and used against the emitter.

## Introduction

Interspecific avian brood parasitism affects the reproductive success of hosts, as brood parasites hijack parental investment causing this to be misdirected towards an unrelated nestling, whilst the hosts’ investment into their own brood is either greatly diminished or lost altogether^1–3^. In order to counter the negative effects of this phenomenon, host species have evolved a range of adaptive traits which can reduce parasitism risk prior to egg-laying (i.e. frontline defenses), as well as during egg-, nestling-, and fledgling stages^1^. Host frontline defenses include secretive female behavior during the breeding season, inaccessible or well-concealed nests, and active nest defense behavior against brood parasites^4,5^. Furthermore, nest defense behavior can also act as a social cue to other potential hosts within the population, greatly enhancing any response against the brood parasite^6,7^ and increasing the hosts’ egg-rejection rates^8^. In turn, brood parasites have evolved traits to bypass these host defenses, leading to a significant variation of responses among brood parasitic study systems^9–14^.

Several studies performed on Nearctic brood parasitic systems address the question regarding the informational value of the host’s activity and behavior for the brood parasites during the nest searching process^9,11,15–17^. While host activity near the nest is essential for the brood parasite to locate the nest^9,11,15^, only two of these studies found a positive correlation between host vocalization (i.e. males singing, and calls uttered near the nest) and the probability of parasitism by cowbirds^16,17^. Brown-headed cowbirds *Molothrus ater* preferred to parasitize willow flycatcher *Empidonax traillii* females with a higher vocalization rate over females that called less often during egg-laying and early incubation^16^. Similarly, higher cowbird parasitism rates were observed at the nests of red-winged blackbird *Agelaius phoeniceus* females that produced more often a call uttered typically when leaving or arriving to the nest, and which was used to coordinate male vigilance and nest defense^17^. In contrast, common cuckoos *Cuculus canorus* exhibited a clear preference to parasitize great reed warblers *Acrocephalus arundinaceus* based on their nest size and nest visibility^12,18^ rather than based on behavioral traits, such as male singing activity and song repertoire^12,13^. To the best of our knowledge, an empirical observation by a Finnish ornithologist originating from 1930, discussed below in detail, constitutes the only description that common cuckoos might rely on a host behavioral trait during host selection or nest searching^19^.

The common cuckoo is one of the most well-studied avian brood parasites^2,20^. Female cuckoos are divided into races (i.e. gentes) that differ with respect to the host species they parasitize, having less than 20 known gentes in Europe^21,22^. Female cuckoos within different gentes are able to recognize their own hosts (e.g. sexually monomorphic, reed-dwelling *Acrocephalus* species with cryptic plumage), and utilize non-primary hosts only if the number of suitable primary host nests is insufficient, despite the striking similarity in appearance between these closely related species^23–26^. Highly accurate species-level host identification was observed also in Edgar Chance’s famous ‘Cuckoo A’, which laid its eggs in tree pipit *Anthus trivialis* nests only if meadow pipit *Anthus pratensis* nests were unavailable, despite the striking degree of similarity between meadow pipits and tree pipits^27^. Therefore, host recognition must be an important fitness component for the cuckoo, given that a mismatch in egg phenotype between hosts and parasites leads to reproductive failure for the cuckoo due to egg rejection^28–30^. Although the recognition of the gent-specific host species and the localization of potential nests is of paramount importance for the fitness of brood parasites, the mechanisms utilized to recognize primary host species and locate their nests remains incompletely understood.

Female cuckoos monitor their potential hosts from vantage points, from where they locate suitable nests to lay their egg in^24^. As cuckoos do not parasitize nests without host activity^31^, some of the host’s behavioral traits are of crucial importance for the brood parasite during the nest searching stage. Also, the nest-site characteristics of hosts are expected to influence the probability of parasitism, and indeed, previous studies have shown that both distance from a vantage point and nest visibility affect the probability of brood parasitism, while vegetation type, cover, nest size, and nest position within the reed has no effect on parasitism rates^18,32,33^. The nest searching strategy utilized by a female might change in accordance with host nest availability: in periods when the abundance of suitable host nests is high, well-concealed nests tend to be less parasitized, while if host nest density is low, cuckoos seemingly invest more effort in nest searching and also parasitize well-hidden nests^34^. Previous studies that have attempted to identify nest localization strategies have focused mainly on visual signals, such as the degree of visibility (e.g. direct nest view, no nest view) of a nest from the nearest cuckoo perch^18,33,34^, while the link between the hosts’ acoustic signals and the ability of brood parasitic cuculids to find suitable host nests has been largely neglected. To the best of our knowledge, only one study was conducted regarding host vocalization and the nest searching strategy of common cuckoos, which found that the conspicuous and easily recognizable song of the male great reed warbler was not a good cue for a female cuckoo when searching for a preferred host’s nest^13^.

The host’s alarm calls, however, are uttered usually close to the nest and could offer important information to a cuckoo regarding the nest’s location. In a study conducted on blackbirds *Turdus merula*, the focal pairs mounted intensive antipredator responses towards magpies *Pica pica* in the close vicinity of their nest (i.e. 1.5 m), but not in cases when the predator was far from the nest (i.e. 6–7 m)^35^. Likewise, willow flycatchers were more likely to defend their nests by chasing away female cowbirds and non-cowbird species when the intruders were close to the nest (i.e. < 2 m), compared to trials when the intruder was not in the close vicinity of the nest (i.e. 2–10 m)^16^. A similar response was also found in three *Acrocephalus* species, which exhibited intensive mobbing and produced alarm calls when their potential predators (i.e. snake, stoat *Mustela erminea*, and marsh harrier *Circus aeruginosus*) were closer to their nests (i.e. 1 m) than in cases when the mounts were placed at a distance of 5 m^36^. Thus, we hypothesize that cuckoos may eavesdrop on host alarm calls to identify the location of their nests, or even elicit them while actively searching for nests if host density is low^34,37^. Recognizing the hosts’ alarming acoustic signals might also enhance the success rate of finding well-hidden nests in inaccessible habitats with tall or dense vegetation (e.g. reed beds). One anecdotal observation from 1930 by Jussi Seppä, a Finnish ornithologist, suggests that common cuckoos use alarm calls to locate suitable host nests much as in the popular children’s game ‘hot and cold’: the closer a cuckoo gets to the correct location of the host’s nest, the more intensive an alarm call will be^19^. Alarm calls might therefore serve as reliable cues for cuckoos in the identification of preferred host nest’s exact location.

To test this hypothesis, we carried out a correlative study and performed two experiments to assess the role of alarm calls uttered by the host and intercepted by the common cuckoo in the nest searching process. Previous studies performed on common cuckoos emphasized on the importance of visual cues such as nest size or the visibility of the nest from the nearest perching site that can be used by the brood parasite to locate the hosts’ nest^18,32,33^. To further corroborate the importance of host alarm calls in the nest searching process of the common cuckoo, we tested how visible the host nests were from the air and from cuckoo perching sites, by performing unmanned aerial vehicle (i.e. drone) flights above parasitized and unparasitized nests.

Based on this hypothesis, we predicted that female cuckoos would respond positively to alarming great reed warblers, seemingly the only host species in our study area^38,39^, by approaching alarming hosts (Experiment 1) or the loudspeaker used for playing back host alarm calls (Experiment 2). Although it is expected that only female common cuckoos search for host nests^40^, some cooperation could exist between the sexes during nest searching. For example, some authors suggested that male cuckoos might play a role in nest searching similarly to other brood parasitic species (e.g. greater spotted cuckoos *Clamator glandarius* and Asian koels *Eudynamys scolopaceus*), where males may provoke hosts, distracting them from the nest while the female lays her egg(s)^41^.

Beside the two experiments, we devised a correlative study to test if the potential interest of the cuckoos towards the host’s alarm calls would lead to an increase in parasitic attempts which would translate into higher parasitism rates in the hosts. Here, we predicted that great reed warblers which mob potential nest predators (i.e. observers perceived as nest predators^42,43^) or brood parasites, unwittingly advertise the proximity of their nests and thus increase their odds of being parasitized.

Also, to estimate the chances of locating host nests based on direct visual cues as suggested by previous studies^18,32,33^, we performed drone flights from the nearest perching site to selected nests, above the nests in mid-air and parallel to the nests on the inner side of the reed bed (i.e. above the water) and measured nest site characteristics (i.e. nest size, distance from reed edges, and vegetation cover)^18^. Here, we predicted that parasitized great reed warbler nests were more visible to human observers on the aerial footage than the unparasitized nests, that are expected to be well concealed in the dense reed. Furthermore, in accordance with a previous study conducted in our study area^18^, we predicted that nests built high above the water surface and with a larger volume are more often parasitized, while nests built further from the inner or outer edge of the reed bed, which have a high vegetation above the cup and are further from cuckoo perches are less likely to be parasitized. We also predicted that host nests that are directly visible from the nearest cuckoo perch are more often parasitized than those that are not visible form the perch.

## Results

### Experiment 1: Cuckoos respond to alarming hosts

**Figure 1:**
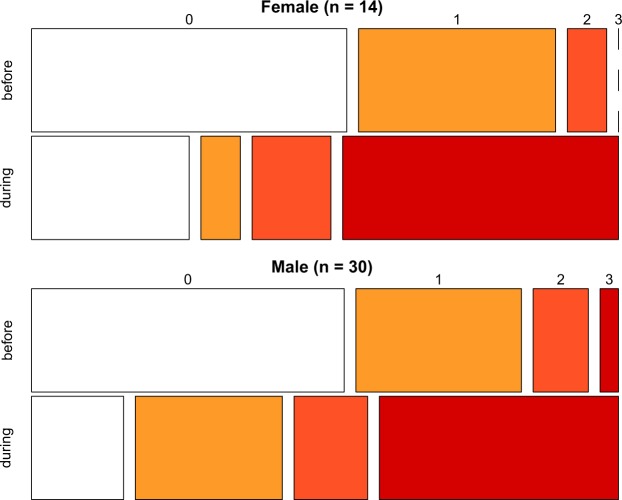
Mosaic plot showing that both female (*n* = 14) and male (*n* = 30) common cuckoos exhibited intensive responses during the 2 minutes when their host, the great reed warbler, engaged in nest defense activities, compared to the 2 minutes prior to the reed warblers alarming. Both female and male cuckoo responses were scored on the following scale: 0 – no response; 1 – typical female or male call within 100 m; 2 – flying towards the alarming great reed warblers; 3 – flying towards the alarming great reed warblers and vocalizing.

To test if common cuckoos were attracted by the alarm calls and mobbing displays (i.e. perching on the top of the reed when alarming and jumping between reed stems) of their hosts, we elicited mobbing reactions from the great reed warblers at their nests, using playback recordings of conspecific alarm calls and a 3D printed cuckoo decoy. During the 51 experimental trials, we recorded 14 cases when a female cuckoo was present and 30 cases when at least one male cuckoo was present. Both male and female cuckoo responses were stronger during the 2 minutes of playback when the great reed warblers were alarming than in the 2-minute pre-alarming period (Fig. 1; females: Fisher’s exact test: *p* = 0.004; males: Fisher’s exact test: *p* < 0.001). We note here that most cuckoos typically flew closer to the mobbing hosts and vocalized, while one male even hovered above the experimental setup for a prolonged period. The number of birds in each response category for both sexes is presented in Table S2 of the Supplementary Material.

### Experiment 2: Cuckoos respond to alarm call playbacks

**Figure 2:**
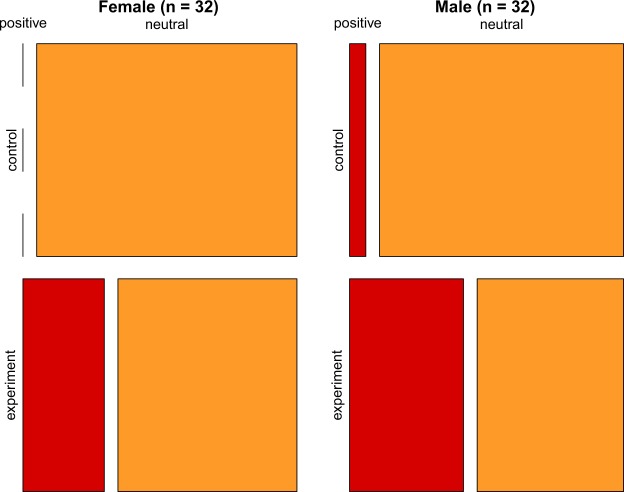
Mosaic plot showing that both female (*n* = 32) and male (*n* = 32) common cuckoos responded positively (i.e. flew closer to the playback device compared to the position where they were initially observed) to the alarm call of great reed warblers by approaching the loudspeaker, compared to the trials when the calls of collared doves were played as a control treatment.

To remove the effect of the host’s visual displays during mobbing, we tested the response of common cuckoos to playbacks of the host’s alarm call and the calls of a neutral control species, the Eurasian collared dove *Streptopelia decaocto*. Female cuckoos approached the speaker more often during the experimental playback trials than during control trials (Fig. 2; 5/16 positive responses in experimental trials and 0/16 in control trials, i.e. no response; Fisher’s exact test: *p* = 0.021). One of the females exhibited a weaker response to the experimental playback compared to the other females, but the statistical results were similar if we treated this response as a neutral reaction (i.e. 4 positive and 12 neutral responses to the experimental playback; Fisher’s exact text: *p* = 0.043). The same result was found for male cuckoos (7/16 positive responses in experimental trials and 1/16 response in controls trials; Fisher’s exact test: *p* = 0.018).

### Correlative study: Alarming hosts experience higher parasitism rate

**Figure 3:**
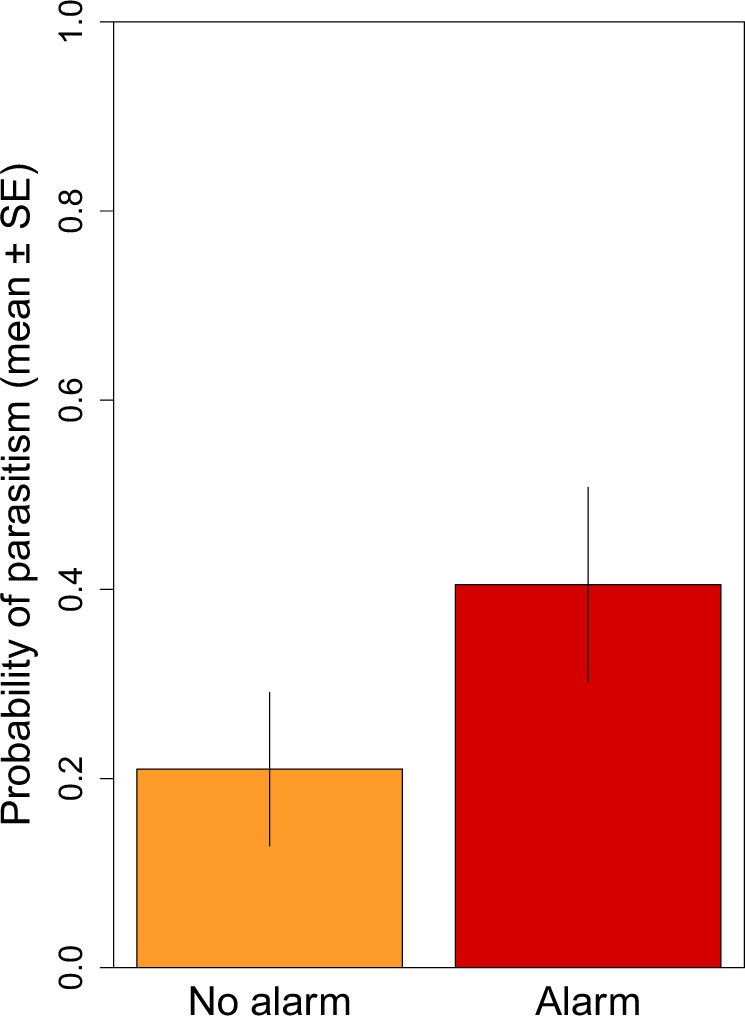
Barplot showing that alarming great reed warblers experience higher probability of brood parasitism by common cuckoos than non-alarming conspecifics. Values indicating the probability of brood parasitism are predicted based on the minimal adequate model (see Table 1) from the correlative study (see main text). Mean predicted values ± standard error (SE) are shown.

To assess the risk great reed warblers expose themselves to by alarming, we quantified parasitism rates and the presence of alarm calls during regular nest visits. The probability of brood parasitism correlated with the presence of host alarm calls directed against human observers. Great reed warblers that uttered alarm calls during regular nest visits encountered a higher probability of parasitism than conspecifics less prone to alarm (Table 1, Fig. 3) and time of the nest visit had a near significant negative effect (Table 1). Laying stage (i.e first 3 days of laying or second 3 days of laying), and the interactions of alarm call presence with time of nest visit, and alarm call presence with laying stage had no effect on the probability of parasitism (full model presented in Table S1 of the Supplementary Material).

**Table 1:** Results of the minimal adequate model showing that alarming great reed warblers were parasitized more often by common cuckoos than conspecifics which were less keen to alarm human observers during regular nest visits.

Fixed effects	Estimate	SE	z value	Wald χ^2^	df	*p*	Variance
(Intercept)	−10.504	1.342	−7.823	61.197	1	<0.001	
Host alarm call	2.748	1.222	2.248	5.054	1	0.024	
Time of nest visit	−0.862	0.457	−1.885	3.551	1	0.059	
**Random effects**							
Site: Nest identity							428.600
Observer identity							0.000

Values for the fixed factor ‘host alarm call’ indicate the difference in the probability of brood parasitism of alarming hosts compared to non-alarming great reed warblers, while ‘time of nest visit’ is a continuous variable, standardized with Z-transformation to mean = 0 and SD = 1, showing the effect of the time of the nest visit on the probability of parasitism. The full model from which the minimal adequate model was derived is presented in Table S1 of the Supplementary Material.

### Drone flights

We performed drone flights to test if visual cues alone are sufficient for the common cuckoo to locate the hosts’ nest (*n* = 16). Contrary to previous results^26,27^, parasitized nests were not more visible than unparasitized nests (0/8 parasitized nests and 1/8 unparasitized nests were visible durint the drone flights; Fisher’s test: *p* = 1.000), but nests closer to the channel’s bank were more likely to be parasitized than nests that were built further from the bank (Wilcoxon rank sum test: W = 50.5, *p* = 0.025). Other nest site characteristics (i.e. nest volume, nest distance from the water, nest height above the water, height of the vegetation above the nest and distance of the nearest cuckoo perch) did not differ significantly between parasitized and unparasitized nests (Table S3 in the Supplementary Material). In general, host nests were well-concealed in the dense reed beds that are characteristic to our study area: only one great reed warbler nest out of 16 was visible on the drone footage. This later nest was unparasitized by cuckoos, despite being built in a narrow reed bed, and being classified in the field as directly visible from the nearest perch.

## Discussion

In this study we tested if brood parasites rely on the alarm calls produced by their hosts, rather than on visual nest searching^18,33,34^ when seeking for nests to lay their eggs in^19^. We found that the nests of the great reed warbler, the host of the brood parasitic common cuckoo in our study area, are hardly visible in the dense reed bed from the nearest cuckoo perches and from mid-air. In contrary, the alarm calls uttered by the hosts are likely to be good nest location cues for the brood parasites, since both female and male common cuckoos exhibited interest towards alarming hosts and alarm call playbacks. Moreover, hosts that were more likely to alarm when approached by human observers during nest visits experienced a higher probability of parasitism.

To the best of our knowledge, our study is the first to provide evidence that brood parasites exploit the alarm calls of their hosts when searching for nests to parasitize, and vigilant hosts expose themselves to the costs of revealing the location of their nests during its defense. Although, if the brood parasites find a host nest it would not necessarily mean that they will parasitize it, the results of our correlative study highlight that the nests of great reed warblers that were prone to alarm at intruders were much more likely to be parasitized by the common cuckoos. An alternative explanation of the results of our correlative study is that the common cuckoos attempted or succeeded to parasitize the great reed warblers in the early stages of the breeding period, which increased the vigilance of the hosts, and thus, increased the probability of alarming at potential threats (i.e. observers during nest visits).

Both female and male common cuckoos exhibited strong interest in host alarm calls by flying closer to the experimental setup and vocalizing, when hosts were present and performed visual displays of aggression (Experiment 1). This interest from the cuckoos was similar also during the playback trials when there was no great reed warbler in sight (Experiment 2), underlining the importance of audio signals compared to visual stimuli of the host. The hosts’ nest defense behavior may also be triggered by male brood parasites, which pose no direct risk for the host, but can provide valuable information for female cuckoos lurking nearby^41,44^.

There are no studies regarding the post-copulatory role played by common cuckoo males in the breeding process. A possible explanation for the unexpected interest of cuckoo males towards the alarm calls of the host parasitized by the cuckoo females might be related to the mating success of males. In a pivotal study regarding the mating system of the common cuckoo performed in Japan, researchers have found that in a study site of 7 km^2^, the vast majority of females mated with only one male, while most males mated only with one or two females^45^. Even more importantly, 65% of the cuckoo males and 50% of the females were not assigned as parents to any of the 136 nestlings sampled. This suggests that some kind of cooperation during the nest searching process between the top female and male cuckoos would result in direct fitness advantages for the individuals with high mating success, as it is in the case other cooperatively breeding brood parasites^3,41^.

Frontline defenses are the most widespread host counter-adaptations against brood parasites^5^: hosts that invest in anti-parasitic defenses (e.g. secretive breeding behavior, well concealed nest) lower their chance of being parasitized, or at least save time and energy to restart the breeding attempt if they are parasitized^43^. These nest defense behaviors usually occur in the close vicinity of the nests^16,35,36^, and are employed against nest predators or brood parasites, sometimes resulting in adverse effects. For example, one recently published study performed on reed warblers and yellow warblers *Setophaga petechia* found that intensive nest defense behavior lowered the risk of brood parasitism only in the case of the reed warblers, but also attracted the attention of predators^14^. Nest defense might also act as a social cue for conspecifics, stimulating them to mount a collective response (e.g. collective mobbing) against the predator or brood parasite, further enhancing its adaptive value^7,42,46^.

Only a few studies dealt with the potential effect of a conspicuous behavior of the host regarding the risk of brood parasitism. These studies found that some conspicuous behaviors, as the calls uttered near the nest, intra-pair signaling or nest-building activity are cues used by brown-headed cowbirds to identify host nests^9,15–17^. Contrary to these, studies performed on common cuckoos and its hosts found that the conspicuous singing of male great reed warblers is neither a reliable nest location cue for the common cuckoo^13^, nor a reliable proxy of the future parental investment of the host^12^. However, neither of these studies examined the effect of alarm calls on the probability of brood parasitism.

If host alarm calls are exploited by brood parasites during nest searching, nest defense as a behavior is likely to survive only if it is adaptive in a different context, rendering the overall benefits higher than its costs. Nest defense can be benefic if it is directed towards a range of enemies: snakes, small carnivores, avian predators posing a risk to the nest (e.g. jays *Garrulus glandarius*, magpies, marsh harriers *Circus aeruginosus*) or to the adult birds (e.g. sparrowhawks *Accipiter nisus*), and brood parasites^14,36,42^. Hosts’ alarm calls act as a social cue and attract the attention of conspecifics from the adjacent territories^47^, which results in a more effective mobbing of the brood parasite^7^ and might increase the probability that a host ejects a parasitic egg^8,46^. Albeit, alarming might be useful for keeping conspecifics or predators at bay^14^, if the alarm calls are uttered near the nest^5,16,35^, alarming might impose the cost of revealing the whereabouts of the host’s nest to eavesdroppers. It is also worth noting, that at sites where the rate of brood parasitism is high^39^, the aggressive behavior towards the cuckoos can be effective only in some cases, the focal nest being successfully parasitized by the brood parasite (or even by multiple brood parasitic individuals) after several parasitism attempts.

An alternative explanation for the persistence of a defensive trait (i.e. alarm calls) acting in a seemingly maladaptive manner (i.e. increasing the likelihood of brood-parasitism) might arise from the breeding ecology of the great reed warbler. The great reed warbler is a typical ‘edge-species’ which prefers to breed on the edge of the reed beds and in the narrow reed-stripes found alongside irrigation channels^47,48^. These high-quality habitats, despite being quickly occupied by early-arriving, large-winged and presumably higher-quality males, are characterized by higher rates of brood parasitism^49–51^. Therefore, irrigation channels function as ecological traps^50,51^ and habitats for sink populations, dependent on the influx of naïve individuals from the habitats with low parasitism rates^52^. These two factors, coupled with the fact that the irrigation channels are artificial habitats created in the past 100 years, prevent natural selection to root out alarm calls, despite the negative fitness consequences suggested by our results.

One implication of our results resides in the potential role of alarm calls in the recognition of the host species by the brood parasites. Passerine nestlings recognize the species-specific alarm calls of their parents^53^, but for brood parasitsic nestlings the imprinting on the alarm calls of their foster parents might be important later in life, when they return to the breeding area as adults. This is supported by a recent study showing that common cuckoos imprinted on their hosts as nestlings, but not on the habitat or nest site where they were raised^31,54^. Some commonly used hosts of the cuckoo live in dense habitats, have cryptic behavior or live in sympatry with other similar species. Nevertheless, female cuckoos tend to recognize and parasitize the host species of their own gentes^23,24^, and parasitize the nest of alternative hosts only if the nests of their main host are in limited supply^2,27^. Based on these findings, it is likely that cuckoos imprint on the alarm call of their foster parents during their nestling or fledgling stages, and they use the alarm calls later during their life to differentiate among hosts similar in appearance, like those belonging to the *Acrocephalus* or *Anthus* genus.

We conclude that common cuckoos can utilize their hosts’ alarm calls when searching for host nests, which represents a novel aspect of the evolutionary arms race between avian brood parasites and their hosts. Common cuckoos eavesdrop on the alarm calls of their host, the great reed warbler, during nest searching, and great reed warblers that alarm more often face higher rates of parasitism. Thus, while alarming plays an important role in keeping nest predators at bay, it can be intercepted by brood parasites, leading to major fitness consequences for both hosts and parasites.

## Methods

### Study area

We performed the different parts of the present study near Apaj village, central Hungary (47.113° N, 19.087° E), between early May and mid-June in 2013, 2014 and 2018. This period coincides with the peak availability (i.e. number) of host nests and the highest rate of parasitism in our study area^18,55^. Here, great reed warblers breed in 1–3 m wide reed beds located along irrigation channels, and experience an unusually high rate of cuckoo parasitism: nests are parasitized in a proportion of 50–70%^39^, compared to the parasitism rates of several hosts ranging between 10–35% at other study sites across the distribution range of the common cuckoo^2,33,56,57^.

All of the work reported here complied with the Hungarian laws and was conducted under the auspices of research permit No. PE/KTF/17190-3/2015 issued by the Middle-Danube-Valley Inspectorate for Environmental Protection, Nature Conservation and Water Management, Budapest.

### Experiment 1: Cuckoos respond to alarming hosts

**Figure 4:**
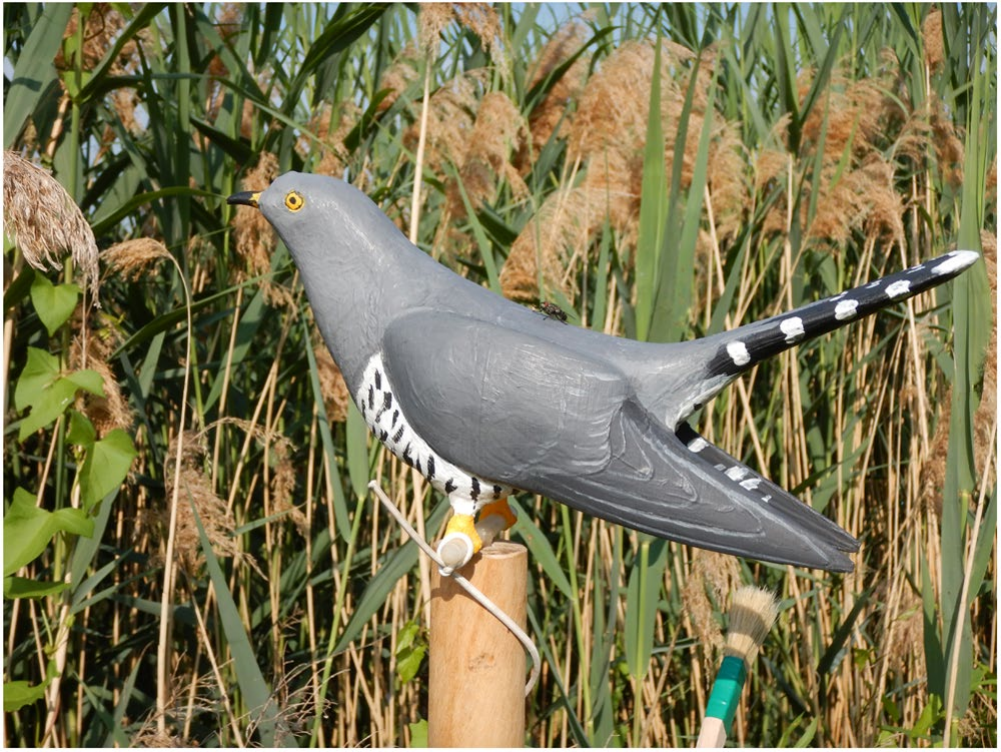
The 3D printed common cuckoo decoy used in our study. The decoy was painted with acrylic paint and mounted on a pole supplied with a noiseless electric motor to stimulate horizontal movement.

We performed an experiment at great reed warbler nests to test if common cuckoos are attracted to the audio (i.e. alarm call) and visual components (i.e. mobbing) of their host’s nest defense behavior. To test our hypothesis, we scored the activity of female and male cuckoos at active great reed warbler nests (*n* = 51) during 2 minutes of silence (i.e. pre-alarming period) and 2 minutes of elicited mobbing by the hosts. We scored the activity of the cuckoos on a 0–3 scale: female and male cuckoo exhibited no reaction (0), produced their characteristic female bubbling call or male call on a perch closer than 100 m (1), exhibited stronger responses by flying silently towards the focal great reed warblers (2), or by flying towards the great reed warblers while producing intense sex-specific calls (3). We performed the trials at great reed warbler nests in the order of which these nests were found, but kept a 500 m distance between nests involved in consecutive trials to minimize the probability of testing the same cuckoos. To elicit mobbing and intense alarming from the focal great reed warbler pair (i.e. intense movement and alarm calls), ensuring both visual and audio stimuli for the cuckoos, we played previously recorded great reed warbler alarm calls using a loudspeaker and a 3D cuckoo decoy bird. We placed the 3D cuckoo decoy at a distance of ca. 0.5 m from the host nest, well concealed by the reed and out of site for any cuckoos, to avoid having a reaction to the 3D decoy instead of the host’s alarm calls and visual displays. This setup ensured the lack of pseudo-replication^58,59^, since the pre-recorded alarm calls (*n* = 10) coupled with the alarm calls of the focal great reed warbler pairs were unique for all trials. The alarm calls were played on a JBL Xtreme loudspeaker (40 W; at about 90 dB, measured by Volcraft SL-100 sound level meter from 1 m distance), while the 3D life-size cuckoo model (313 × 92 × 200 mm) supplied by 3D QuickPrinting UK was printed with an Ultimaker 2+ 3D printer using transparent Ultimaker PLA filament. We painted the transparent 3D cuckoo with acrylics to resemble a cuckoo, and fitted it on a standing support supplied with a silent robotic motor to stimulate the display movements of cuckoos (Fig. 4., additional video in the Supplementary Material). Experiments were carried out by two observers (AM and MB).

### Experiment 2: Cuckoos respond to alarm call playbacks

During our first experiment, perching common cuckoos were exposed to both the sound and sight of their hosts in the vicinity of the nest, but the informational value of the two types of stimuli was unknown. To test if cuckoos truly responded to the alarm calls uttered by their hosts and not to their aggressive displays, we carried out a second experiment. In this second experiment, we played host alarm calls (*n* = 16) and collared dove calls as a neutral control (*n* = 16) in a random order, at 32 different sites where female cuckoos were heard (i.e. the presence of individuals was confirmed). Cuckoos were observed usually at distance of 40–50 m, and females were identified based on their typical bubbling call^40^. The playback begun within 2 minutes after the female cuckoo was heard, lasted for 2 minutes and was followed by a 2 minute post-playback period, while the movements of both female and male cuckoos were quantified. A response was considered positive if a focal bird approached the playback device compared to the distance it was initially observed, and neutral if it did not react or increased their distance (i.e. flew away) from the playback device compared to their initial position. Both female and male cuckoos are typically territorial in the breeding season, and previous studies with VHS and GPS telemetry typically revealed less than 1 km long, partly overlapping cuckoo territories along the channels in our study area^44,60,61^. Thus, in order to reduce potential pseudo-replication^58,59^ in our data, we used every sound file only once (both experimental and control files) at sites at least 2 km apart. The great reed warbler alarm calls (*n* = 16) and collared dove calls (*n* = 16) were recorded during the breeding season in 2018 and each of them was played only once, using a JBL Xtreme loudspeaker (40 W), at about 90 dB. All trials were performed by CM.

### Correlative study: Alarming hosts experience higher parasitism rates

We collected data on the breeding performance and alarm calls of great reed warblers, a facultatively polygynous^42,62^ host widely used by cuckoos across several parts of Europe^21,22,39,63,64^.

We visited great reed warbler nests (*n* = 170) in the egg-laying stage on multiple occasions (*n* = 330 visits in total, mean number of visits ± SD per nest: 1.94 ± 1.10) and quantified clutch initiation date (i.e. day 0 = laying date of the first egg), clutch size (i.e. sum of great reed warbler and cuckoo eggs at the time of each visit), and the reaction (i.e. alarm calls) of hosts to the human observer, as an indicator of nest defense intensity. Great reed warblers respond aggressively to human intrusion similarly to the intrusion of cuckoos or nest predators^42,56^ and produce a typical alarming call. Alarming behavior of hosts during nest visits was assessed by four observers on a binary scale (0: no alarm call, 1: at least one great reed warbler alarmed at the nest). All four observers (AM, MB and two field assistants) were trained to recognize the great reed warbler alarm calls prior to the observations and all nest visits were performed in the morning hours, between 6:00 AM and 11:00 AM, covering the peak activity period of great reed warblers^16^.

### Drone flights

Previous studies^18,32,33^ show that cuckoos perch on trees serving as vantage points, when searching for suitable host nests. To check whether nests are visible from such vantage points or from above the reed, we conducted drone flights above 8 parasitized and 8 unparasitized randomly chosen focal nests from one of the channels in our study site. Flights were carried out with a DJI Phantom 4 drone set to record aerial videos at 4k resolution and 25 fps, based on the following protocol, simulating cuckoo flights: first, the drone ascended to 6–8 m (i.e. depending on the height of the closest cuckoo vantage point), then performed a slow descent approaching the focal nest, until reaching the area above the nest (i.e. 1 m above the reed bed). After this operation, the drone descended to the inner side of the reed bed and hovered above the water in order to get a clear view of the reed bed from the inner side. While performing the drone flights, the exact location of the nest was pointed out with a 1 m long ruler by one observer (AM or MB) standing in the reed near the focal nest.

Nest volume (expressed in cm^3^ as the volume of a cylinder with the height equal to the nest’s height and its base diameter equal to width of the nest), and the following nest site characteristics were measured similarly as described in a previous study^18^: nest height above the water surface (cm), nest distance from the bank and from open water (cm), vegetation height above nest (cm) and nest visibility from the nearest cuckoo perch (scored on a three-level factor: direct nest view, indirect nest view or no nest view). The recorded videos were screened for nests using VLC media player (v. 3.0.8. Vetinari) at normal speed by an assistant person with no previous knowledge about the aims of the study to ensure the blind evaluation of the recordings, and subsequently by the first author of the study, rendering the same results.

### Statistical analyses

We hypothesized that cuckoos would exhibit a stronger response during the alarming period compared to the pre-alarming period (see above). Thus, the data from Experiment 1 were analyzed using one-tailed Fisher’s exact test^63^. Prior to the analysis, we excluded all trials in which no cuckoo responses were recorded neither before, nor during the alarming period.

Data from Experiment 2 were analyzed with one-tailed Fisher’s exact test^65^, due to our hypotheses that cuckoos would mount a stronger response to the alarm calls compared to the control calls.

Data gathered during our correlative study were analyzed using a generalized linear mixed-effect model (GLMM) with binomial error distribution^66^. Host alarm calls might have higher informational value for the brood parasite in the first days of laying of the host, which could ensure brood parasites that their progeny would hatch before those of the hosts^67^ or of the concurrent cuckoos, as it is the case at our study site, where multiple parasitism is common^39^. For the Eurasian reed warblers, a close relative of the great reed warbler, the first three days of laying clearly represents a high-risk period, but later on in the laying period the risk is reduced^2^. Therefore, we divided the egg-laying period into two subperiods forming two groups: “high risk” (i.e. first 3 days of laying) and “low risk” (i.e. the second 3 days of laying) groups.

We entered the occurrence of brood parasitism of individual nests at every visit as a binary response variable (0: no cuckoo eggs in the nest, 1: at least one cuckoo egg in the nest) in the model. The presence of absence of host alarm calls (factor with two levels), risk of parasitism (factor with two levels), time of visit within the day (expressed in number of minutes elapsed from midnight, standardized with Z-transformation to mean = 0 and SD = 1 to improve model convergence), and the second-order interactions between the presence of alarm call and the variables listed above were included as fixed terms in the model. Observer ID and nest ID nested under site ID (i.e. different irrigation channels hosting the great reed warblers) were all included as random terms. After constructing the full model containing all the predictors listed above and their interactions, we simplified the model utilizing a stepwise backwards elimination procedure based on the significance level (*p* < 0.050) of the predictors, in each step dropping the predictor with the highest *p* value, until reaching the minimal adequate model containing only significant or marginally significant (*p* < 0.100) effects. We tested the full and the minimum adequate model for multicollinearity between predictors using the ‘*vif.mer*’ function^66,68^, which calculates the variance inflation factor (VIF) for each predictor separately. Since VIF was less than 2.09 for all variables for the full model and 1.12 for the variables in the minimal model, we concluded that there was no multicollinearity between predictors. The GLMM were performed using the function ‘*glmer*’, from the R package ‘*lme4’*^69^, using Laplace approximation for parameter estimation^70^ and ‘*bobyqa’* algorithm for model optimization^71^. Data handling, date and time conversions were performed using the packages ‘*chron’*^72^ and ‘*doBy*’^73^ in the R statistical environment^65^.

The numerical data collected during the drone study, namely the nest site characteristics (i.e. nest volume, nest distance from the bank and from the water, nest height of above the water surface, height of the reed above the nest cup and the distance to the nearest cuckoo perch) of parasitized and unparasitized nests were analyzed using one-tailed Wilcoxon rank sum tests^74^ in accordance with the predicted direction for each variable presented in the Introduction and summarised in Table S4 of the Supplementary Material, while the nest view expressed in a three-level factor (i.e. direct nest view, indirect nest view and no nest view) was analyzed using one-tailed Fisher’s exact test^65^.

All statistical analyses were conducted in RStudio version 1.0.153^71^, running R version 3.3.3^75^.

## Supplementary information

Great reed warblers mobbing 3D printed cuckoo decoy

Supplementary Material

R markdown script

Correlative study data

Drone study data

Experimental study data

## Data Availability

All data from this study are included in this publication and its Supplementary Material.
