# Supplementary material for: Host alarm calls attract the unwanted attention of the brood parasitic common cuckoo
Source: Sci Rep. 2019 Dec 6;9:18563. doi: 10.1038/s41598-019-54909-1 (PMC6898711; doi:10.1038/s41598-019-54909-1)
Supplement: Supplementary file 2 — Supplementary Material [file 41598_2019_54909_MOESM2_ESM.pdf]

# Host alarm calls attract the unwanted attention of the brood parasitic common cuckoo

# – Supplementary Material –


**Attila Marton[1,2*], Attila Fülöp[1,2,3], Katalin Ozogány[1], Csaba Moskát[4,†] and Miklós Bán[1,3,†]**

[1] Department of Evolutionary Zoology and Human Biology, University of Debrecen, Debrecen, Hungary

[2] Juhász-Nagy Pál Doctoral School, University of Debrecen, Debrecen, Hungary

[3] MTA-DE Behavioural Ecology Research Group, Department of Evolutionary Zoology and Human Biology, University of Debrecen, Debrecen, Hungary

[4] MTA-ELTE-MTM Ecology Research Group, a joint research group of the Hungarian Academy of Sciences, the Biological Institute of the Eötvös Loránd University and the Hungarian Natural History Museum, Budapest, Hungary

[†] joint senior authorship

*** Correspondence**:

Attila Marton

Department of Evolutionary Zoology and Human Biology, University of Debrecen

Egyetem tér 1, H-4032 Debrecen, Hungary

Email: martonattila2010@gmail.com

Phone number: 0040725533086

**Abstract**

It is well known that avian brood parasites lay their eggs in the nests of other bird species, called hosts. It remains less clear, however, just how parasites are able to recognize their hosts and identify the exact location of the appropriate nests to lay their egg in. While previous studies attributed high importance to visual signals in finding the hosts' nests (e.g. nest building activity or the distance and direct sight of the nest from vantage points used by the brood parasites), the role of host acoustic signals during the nest searching stage has been largely neglected. We present experimental evidence that both female and 35 common cuckoos *Cuculus canorus* pay attention to their host's, the great reed warbler *Acrocephalus arundinaceus* alarm calls, relative to the calls of an unparasitized species used as controls. Parallel to this, we found no difference between the visibility of parasitized and unparasitized nests during drone flights, but great reed warblers that alarmed more frequently experienced higher rates of parasitism. We conclude that alarm calls might be advantageous for the hosts when used against enemies or for alerting conspecifics, but can act in a detrimental manner by providing important nest location cues for eavesdropping brood parasites. Our results suggest that host alarm calls may constitute a suitable trait on which cuckoo nestlings can imprint on to recognize their primary host species later in life. Our study contributes to the growing body of knowledge regarding the context-dependency of animal signals, by providing a novel example of a beneficial acoustic trait intercepted by a heterospecific and used against the emitter.

**This supplementary material contains:**

- Table S1: Results of the full model showing all of the predictors measured of the probability of brood parasitism of great reed warbler *Acrocephalus arundinaceus* nests by common cuckoos *Cuculus canorus*.

- Table S2: Contingency table with the responses of female and male common cuckoos *Cuculus canorus* in the 2 minutes prior and 2 minutes during the mobbing display and alarm calls of their host.

- Table S3: Contingency table with the number of individual female and male common cuckoos *Cuculus canorus* which approached the playback device playing their host' alarm call or the call of the collared dove *Streptopelia* decaocto, used as a control.

- Table S4: Measured nest site characteristics and their predicted effect on brood parasitism (as described in Moskát and Honza 2000).

- data regarding the correlative study, experiments and drone flights in csv formats

- R markdown script of the data analyses in html format

- sample video of great reed warblers mobbing the 3D cuckoo decoy

**Table S1.** Results of the full model showing all the potential predictors of the probability of brood parasitism of great reed warbler nests by common cuckoos. Values for the fixed factor, 'alarm call', indicate the difference of alarming hosts compared to non-alarming great reed warblers, 'laying stage' is a two-level factor of nests in their first 3 days of egg laying or second 3 days of egg laying, 'time of nest visit' is a scaled continuous variable which measures the number of elapsed minutes since midnight standardized with a Z transformation (mean = 0 and SD = 1).

| | Estimate | SE | z value | Wald $\chi^2$ | df | *p* | Variance |
|---|---|---|---|---|---|---|---|
| **FIXED EFFECTS** | | | | | | | |
| **(Intercept)** | -10.746 | 1.474 | -7.286 | 53.089 | 1 | <0.001 | |
| **Alarm call** | 3.543 | 2.199 | 1.611 | 2.595 | 1 | 0.107 | |
| **Laying stage** | 0,306 | 1.255 | 0.244 | 0.059 | 1 | 0.870 | |
| **Time of nest visit** | -1.025 | 0,539 | -1.903 | 3.620 | 1 | 0.057 | |
| **Alarm call × Laying stage** | -1.561 | 2.480 | -0.630 | 0.529 | 1 | 0.529 | |
| **Alarm call × Time of nest visit** | 1.151 | 1.346 | 0.855 | 0.730 | 1 | 0.392 | |
| | | | | | | | |
| **RANDOM EFFECTS** | | | | | | | |
| **Site : Nest identity** | | | | | | | 439.700 |
| **Observer identity** | | | | | | | 0.000 |

**Table S2.** Responses of female and male common cuckoos in the 2 minutes prior and during the 2 minutes of alarm calls and mobbing display of their host, the great reed warbler. The response of the cuckoos was scored on a four-level scale, as follows: 0 - no reaction; 1- characteristic female bubbling call or male call on a perch closer than 100 m; 2 - flight towards the great reed warbler nest; 3 - flight towards the nest coupled with characteristic female or male call.

| Sex | Group | 0 | 1 | 2 | 3 |
|---|---|---|---|---|---|
| *females* | before playback | 8 | 5 | 1 | 0 |
| | during playback | 4 | 1 | 2 | 7 |
| *males* | before playback | 17 | 9 | 3 | 1 |
| | during playback | 5 | 8 | 4 | 13 |

**Table S3.** Number individual female and male common cuckoos which approached the playback device playing their host' alarm call (experimental trials; n = 16) and the sound of the collared dove (control trials; n = 16). The response of the cuckoos was considered positive if they approached the playback device compared to the distance they were initially observed, and neutral if the focal birds did not react or increased their distance (i.e. flew away) from the playback device compared to their initial position.

| Sex | Response | Experimental trials | Control trials |
|---|---|---|---|
| *females* | positive | 5 | 0 |
| | neutral | 11 | 16 |
| *males* | positive | 7 | 1 |
| | neutral | 9 | 15 |

**Table S4.** Measured nest site characteristics and their predicted effect on brood parasitism (as described in Moskát and Honza 2000) of non-parasitized (n = 8) and parasitized (n = 8) great reed warbler nests. Nest volume was calculated as the volume of a cylinder with of the height of the nest (in cm) and base diameter equal to the width of the nest (in cm).

| | | Non-parasitized | | | | | Parasitized | | | | | |
|---|---|---|---|---|---|---|---|---|---|---|---|---|
| **Fixed effect** | **Predicted effect on parasitism** | **Mean** | **SD** | **CV** | **Median** | **Range** | **Mean** | **SD** | **CV** | **Median** | **Range** | ***p*** |
| Nest volume ($cm^3$) | positive | 1184.4 | 488.6 | 0.41 | 1059.75 | 785-2279 | 1241.77 | 400.59 | 0.32 | 1127.65 | 763-1899 | 0.370 |
| Distance from water (cm) | negative | 70 | 20.35 | 0.29 | 62.5 | 50-110 | 78.12 | 26.98 | 0.34 | 80 | 40-115 | 0.739 |
| Distance from bank (cm) | negative | 238.75 | 72.19 | 0.3 | 245 | 100-320 | 178.12 | 39.27 | 0.22 | 170 | 125-240 | 0.025 |
| Nest height above water (cm) | positive | 82.87 | 29.12 | 0.35 | 83.5 | 40-120 | 79.62 | 25.76 | 0.32 | 82.5 | 42-118 | 0.571 |
| Vegetation height above nest (cm) | negative | 178.75 | 25.31 | 0.14 | 190 | 140-200 | 200 | 45.66 | 0.22 | 200 | 140-280 | 0.853 |
| Distance from closest perch (m) | positive | 11.75 | 5.57 | 0.47 | 12 | 3-20 | 10.75 | 6.92 | 0.64 | 8 | 5-25 | 0.688 |

## Bibliography

Moskát C, Honza M. 2000. Effect of nest and nest site characteristics on the risk of cuckoo Cuculus canorus parasitism in the great reed warbler Acrocephalus arundinaceus. Ecography 23:335–341.
