# Supplementary material for: Host alarm calls attract the unwanted attention of the brood parasitic common cuckoo
Source: Sci Rep. 2019 Dec 6;9:18563. doi: 10.1038/s41598-019-54909-1 (PMC6898711; doi:10.1038/s41598-019-54909-1)
Supplement: Supplementary file 3 — R markdown script [file 41598_2019_54909_MOESM3_ESM.pdf]

Host alarm calls attract the unwanted attention of the brood parasitic common cuckoo

Attila Marton, Attila FĂĽlĂ¶p, Katalin OzogĂˇny, Csaba MoskĂˇt and MiklĂłs BĂˇn

14 November 2019

Files from the Supplementary Material needed to run the code:

1. Correlative-study-data.csv

- nestid: a concatenated id showing the number of given nest in given year and the year
- site: name of channel (Arapaszto, Apaj, DVCS)
- parasite: presence of parasitism, 1 if cc.eggs > 1
- p.risk: risk of parasitism, “high” in the first 3 days of laying, “low” in the second 3 days of laying
- b.pair: binomial variable of presence/absence of alarming at nest visits
- observer: initials of observers
- s.mminutes: mminutes Z-transformed to mean = 0 and SD = 1

2. Experiment1-data.csv

- date: format yyyy.mm.dd
- time: format hh:mm
- before.ccm: male common cuckoo activity near the focal great reed warbler nest 2 minutes prior to alarm call (0 = none, 1 = calling, 2 = flying closer, 3 = flying closer and vocalizing intensively)
- during.ccm: male common cuckoo activity near the focal great reed warbler nest during 2 minutes of alarm call (0 = none, 1 = calling, 2 = flying closer, 3 = flying closer and vocalizing intensively)
- before.ccf: female common cuckoo activity near the focal great reed warbler nest 2 minutes prior to alarm call (0 = none, 1 = calling, 2 = flying closer, 3 = flying closer and vocalizing intensively)
- during.ccf: female common cuckoo activity near the focal great reed warbler nest during 2 minutes of alarm call (0 = none, 1 = calling, 2 = flying closer, 3 = flying closer and vocalizing intensively)
- observer: initials of observers

3. Drone-study-data.csv

Create PDF in your applications with the Pdfcrowd HTML to PDF API PDFCROWD

- nestid: a concatenated id showing the number of given nest in given year and the year
- id: the id number of given nest within given year
- year: the year of the study
- date: format yyyy.mm.dd
- parasite: presence of parasitism, 1 if cc.eggs > 1
- nest.height: the height of the nest from top to bottom in cm
- nest.width: the width of the nest at the top in cm
- dist.water: distance of nest from water in cm
- dist.bank: distance of nest from the bank in cm
- above.water: distance between water and the top of the nest in cm
- above.nest: distance from the top of the nest and the top of the vegetation in cm
- visibility: direct nest view (DNV), indirect nest view (INV), no nest view (NNW) from the nest to the nearest perching tree
- perch.dist: distance in m to the nearest perching tree
- observer: initials of observers

```
#library(Matrix)
#library(lme4)
library(lsmeans)
#library(car)
#library(exactRankTests)
library("knitr")
```

```
#source: https://github.com/aufrank/R-hacks/blob/master/mer-utils.R

vif.mer <- function (fit) {
    ## adapted from rms::vif

    v <- vcov(fit)
    nam <- names(fixef(fit))

    ## exclude intercepts
    ns <- sum(1 * (nam == "Intercept" | nam == "(Intercept)"))
    if (ns > 0) {
        v <- v[-(1:ns), -(1:ns), drop = FALSE]
        nam <- nam[-(1:ns)]
```

Create PDF in your applications with the Pdfcrowd HTML to PDF API PDFCROWD

```
    }

    d <- diag(v)^0.5
    v <- diag(solve(v/(d %o% d)))
    names(v) <- nam
    v
}
```

```
r0 = "#FFFFFF"
r1 = "#FD9A28"
r2 = "#FF5126"
r3 = "#d50000"
```

```
df <- read.csv("Correlative-study-data.csv", header = T, sep = ";", dec = ".", na.strings = c("NA", "", " "))
summary(df)
```

```
##        nr                nestid       mminutes                   site
##  Min.   :  1.00   2013_275:  5   Min.   :345.0   Apaj             :117
##  1st Qu.: 83.25   2013_309:  5   1st Qu.:546.2   Arapaszto        : 47
##  Median :165.50   2013_311:  5   Median :595.0   DVCS             :131
##  Mean   :165.50   2013_313:  5   Mean   :582.5   Kunszentmiklos: 35
##  3rd Qu.:247.75   2013_34 :  5   3rd Qu.:626.5
##  Max.   :330.00   2013_39 :  5   Max.   :660.0
##                   (Other) :300
##     parasite        p.risk        b.pair       observer    s.mminutes
##  Min.   :0.0000   high:158   Min.   :0.0000   BM : 83   Min.   :-4.1857
##  1st Qu.:0.0000   low :172   1st Qu.:0.0000   GN :127   1st Qu.:-0.6392
##  Median :0.0000              Median :0.0000   MA : 15   Median : 0.2198
##  Mean   :0.2485              Mean   :0.1727   ZSI:105   Mean   : 0.0000
##  3rd Qu.:0.0000              3rd Qu.:0.0000             3rd Qu.: 0.7749
##  Max.   :1.0000              Max.   :1.0000             Max.   : 1.3653
##
```

```
str(df)
```

Create PDF in your applications with the Pdfcrowd HTML to PDF API PDFCROWD

```
## 'data.frame':    330 obs. of  9 variables:
##  $ nr        : int  1 2 3 4 5 6 7 8 9 10 ...
##  $ nestid    : Factor w/ 170 levels "2013_104","2013_106",..: 1 2 2 2 2 3 3 4 4 5 ...
##  $ mminutes  : int  480 530 600 615 570 545 630 540 540 634 ...
##  $ site      : Factor w/ 4 levels "Apaj","Arapaszto",..: 1 1 1 1 1 1 1 1 1 1 ...
##  $ parasite  : int  1 0 0 0 0 0 0 0 0 0 ...
##  $ p.risk    : Factor w/ 2 levels "high","low": 2 1 1 2 2 1 2 1 2 1 ...
##  $ b.pair    : int  0 0 0 0 0 0 0 0 0 0 ...
##  $ observer  : Factor w/ 4 levels "BM","GN","MA",..: 1 1 4 4 4 1 1 1 1 1 ...
##  $ s.mminutes: num  -1.807 -0.926 0.308 0.572 -0.221 ...
```

```
df$nestid     <- as.factor(df$nestid)
df$site       <- as.factor(df$site)
df$observer   <- as.factor(df$observer)
df$p.risk     <- as.factor(df$p.risk)
df$b.pair     <- as.factor(df$b.pair)
df$s.mminutes <- scale(df$mminutes)
df$parasite   <- as.numeric(df$parasite)

# glmer model

p_b.p <- glmer(parasite ~ b.pair
              + p.risk
              + s.mminutes
              + b.pair:p.risk
              + b.pair:s.mminutes
              + (1|site:nestid)
              + (1|observer)
              ,
              data=df,
              family = "binomial",
              control = glmerControl(optimizer = "bobyqa",
                        optCtrl = list(maxfun = 500000)),
              na.action = na.omit)
```

Create PDF in your applications with the Pdfcrowd HTML to PDF API PDFCROWD

```
## singular fit
```

```
summary(p_b.p)
```

```
## Generalized linear mixed model fit by maximum likelihood (Laplace
##   Approximation) [glmerMod]
##  Family: binomial  ( logit )
## Formula:
## parasite ~ b.pair + p.risk + s.mminutes + b.pair:p.risk + b.pair:s.mminutes +
##     (1 | site:nestid) + (1 | observer)
##    Data: df
## Control:
## glmerControl(optimizer = "bobyqa", optCtrl = list(maxfun = 5e+05))
##
##      AIC      BIC   logLik deviance df.resid
##    245.4    275.8   -114.7    229.4      322
##
## Scaled residuals:
##      Min       1Q   Median       3Q      Max
## -1.20123 -0.00714 -0.00393 -0.00220  2.94854
##
## Random effects:
##  Groups      Name        Variance Std.Dev.
##  site:nestid (Intercept) 439.7    20.97
##  observer    (Intercept)   0.0     0.00
## Number of obs: 330, groups:  site:nestid, 170; observer, 4
##
## Fixed effects:
##                     Estimate Std. Error z value Pr(>|z|)
## (Intercept)         -10.7462     1.4751  -7.285 3.21e-13 ***
## b.pair1               3.5434     2.1993   1.611   0.1072
## p.risklow             0.3064     1.2550   0.244   0.8071
## s.mminutes           -1.0257     0.5391  -1.903   0.0571 .
## b.pair1:p.risklow    -1.5615     2.4804  -0.630   0.5290
## b.pair1:s.mminutes   1.1510     1.3464   0.855   0.3926
```

Create PDF in your applications with the Pdfcrowd HTML to PDF API PDFCROWD

```
## ---
## Signif. codes:  0 '***' 0.001 '**' 0.01 '*' 0.05 '.' 0.1 ' ' 1
##
## Correlation of Fixed Effects:
##             (Intr) b.par1 p.rskl s.mmnt b.pr1:p.
## b.pair1     -0.360
## p.risklow   -0.375  0.215
## s.mminutes   0.240 -0.089  0.270
## b.pr1:p.rsk  0.175 -0.773 -0.497 -0.125
## b.pr1:s.mmn -0.117 -0.268 -0.110 -0.405  0.167
## convergence code: 0
## singular fit
```

```
Anova(p_b.p, type = "III")
```

```
## Error in Anova(p_b.p, type = "III"): could not find function "Anova"
```

```
drop1(p_b.p, test = "Chisq")
```

```
## singular fit
## singular fit
```

```
## Single term deletions
##
## Model:
## parasite ~ b.pair + p.risk + s.mminutes + b.pair:p.risk + b.pair:s.mminutes +
##     (1 | site:nestid) + (1 | observer)
##                   Df    AIC     LRT Pr(Chi)
## <none>               245.42
## b.pair:p.risk      1 243.82 0.40642  0.5238
## b.pair:s.mminutes  1 244.16 0.74743  0.3873
```

Create PDF in your applications with the Pdfcrowd HTML to PDF API PDFCROWD

```
p_b.p_2 <- update(p_b.p, .~.
                    - b.pair:p.risk
                    )
```

```
## singular fit
```

```
summary(p_b.p_2)
```

```
## Generalized linear mixed model fit by maximum likelihood (Laplace
##   Approximation) [glmerMod]
##  Family: binomial  ( logit )
## Formula: parasite ~ b.pair + p.risk + s.mminutes + (1 | site:nestid) +
##     (1 | observer) + b.pair:s.mminutes
##    Data: df
## Control:
## glmerControl(optimizer = "bobyqa", optCtrl = list(maxfun = 5e+05))
##
##      AIC      BIC   logLik deviance df.resid
##    243.8    270.4   -114.9    229.8      323
##
## Scaled residuals:
##      Min       1Q   Median       3Q      Max
## -1.20654 -0.00691 -0.00387 -0.00221  2.53906
##
## Random effects:
##  Groups      Name        Variance  Std.Dev.
##  site:nestid (Intercept) 4.437e+02 2.106e+01
##  observer    (Intercept) 7.526e-14 2.743e-07
## Number of obs: 330, groups:  site:nestid, 170; observer, 4
##
## Fixed effects:
##                      Estimate Std. Error z value Pr(>|z|)
## (Intercept)          -10.6288     1.4402  -7.380 1.58e-13 ***
## b.pair1                2.5011     1.4290   1.750   0.0801 .
```

Create PDF in your applications with the Pdfcrowd HTML to PDF API PDFCROWD

```
## p.risklow           -0.1040     1.0932  -0.095   0.9242
## s.mminutes          -1.0809     0.5473  -1.975   0.0483 *
## b.pair1:s.mminutes   1.3252     1.3080   1.013   0.3110
## ---
## Signif. codes:  0 '***' 0.001 '**' 0.01 '*' 0.05 '.' 0.1 ' ' 1
##
## Correlation of Fixed Effects:
##             (Intr) b.par1 p.rskl s.mmnt
## b.pair1     -0.382
## p.risklow   -0.268 -0.370
## s.mminutes   0.293 -0.329  0.273
## b.pr1:s.mmn -0.170 -0.228 -0.021 -0.401
## convergence code: 0
## singular fit
```

```
drop1(p_b.p_2, test = "Chisq")
```

```
## singular fit
## singular fit
```

```
## Single term deletions
##
## Model:
## parasite ~ b.pair + p.risk + s.mminutes + (1 | site:nestid) +
##     (1 | observer) + b.pair:s.mminutes
##                    Df    AIC     LRT Pr(Chi)
## <none>                243.82
## p.risk              1 241.83 0.00893  0.9247
## b.pair:s.mminutes   1 242.93 1.10998  0.2921
```

```
p_b.p_3 <- update(p_b.p_2, .~.
                    - p.risk
                  )
```

Create PDF in your applications with the Pdfcrowd HTML to PDF API PDFCROWD

```
## singular fit
```

```
summary(p_b.p_3)
```

```
## Generalized linear mixed model fit by maximum likelihood (Laplace
##   Approximation) [glmerMod]
##  Family: binomial  ( logit )
## Formula:
## parasite ~ b.pair + s.mminutes + (1 | site:nestid) + (1 | observer) +
##     b.pair:s.mminutes
##    Data: df
## Control:
## glmerControl(optimizer = "bobyqa", optCtrl = list(maxfun = 5e+05))
##
##      AIC      BIC   logLik deviance df.resid
##    241.8    264.6   -114.9    229.8      324
##
## Scaled residuals:
##      Min       1Q   Median       3Q      Max
## -1.20321 -0.00697 -0.00391 -0.00219  2.63570
##
## Random effects:
##  Groups      Name        Variance Std.Dev.
##  site:nestid (Intercept) 443.7    21.06
##  observer    (Intercept)   0.0     0.00
## Number of obs: 330, groups:  site:nestid, 170; observer, 4
##
## Fixed effects:
##                     Estimate Std. Error z value Pr(>|z|)
## (Intercept)        -10.6679     1.3850  -7.702 1.34e-14 ***
## b.pair1              2.4521     1.3279   1.847   0.0648 .
## s.mminutes          -1.0671     0.5249  -2.033   0.0421 *
## b.pair1:s.mminutes   1.3233     1.3094   1.011   0.3122
## ---
## Signif. codes:  0 '***' 0.001 '**' 0.01 '*' 0.05 '.' 0.1 ' ' 1
```

Create PDF in your applications with the Pdfcrowd HTML to PDF API PDFCROWD

```
##
## Correlation of Fixed Effects:
##             (Intr) b.par1 s.mmnt
## b.pair1     -0.536
## s.mminutes   0.393 -0.253
## b.pr1:s.mmn -0.181 -0.258 -0.409
## convergence code: 0
## singular fit
```

```
drop1(p_b.p_3, test = "Chisq")
```

```
## singular fit
```

```
## Single term deletions
##
## Model:
## parasite ~ b.pair + s.mminutes + (1 | site:nestid) + (1 | observer) +
##     b.pair:s.mminutes
##                   Df    AIC    LRT Pr(Chi)
## <none>               241.83
## b.pair:s.mminutes  1 240.94 1.1065  0.2929
```

```
p_b.p_4 <- update(p_b.p_3, .~.
                    - b.pair:s.mminutes
                  )
```

```
## singular fit
```

```
summary(p_b.p_4)
```

```
## Generalized linear mixed model fit by maximum likelihood (Laplace
##   Approximation) [glmerMod]
```

Create PDF in your applications with the Pdfcrowd HTML to PDF API PDFCROWD

```
##  Family: binomial  ( logit )
## Formula:
## parasite ~ b.pair + s.mminutes + (1 | site:nestid) + (1 | observer)
##    Data: df
## Control:
## glmerControl(optimizer = "bobyqa", optCtrl = list(maxfun = 5e+05))
##
##      AIC      BIC   logLik deviance df.resid
##    240.9    259.9   -115.5    230.9      325
##
## Scaled residuals:
##      Min       1Q   Median       3Q      Max
## -1.15549 -0.00704 -0.00435 -0.00264  2.42076
##
## Random effects:
##  Groups      Name        Variance Std.Dev.
##  site:nestid (Intercept) 428.6    20.7
##  observer    (Intercept)   0.0     0.0
## Number of obs: 330, groups:  site:nestid, 170; observer, 4
##
## Fixed effects:
##             Estimate Std. Error z value Pr(>|z|)
## (Intercept) -10.5043     1.3429  -7.822  5.2e-15 ***
## b.pair1       2.7488     1.2227   2.248   0.0246 *
## s.mminutes   -0.8621     0.4575  -1.885   0.0595 .
## ---
## Signif. codes:  0 '***' 0.001 '**' 0.01 '*' 0.05 '.' 0.1 ' ' 1
##
## Correlation of Fixed Effects:
##            (Intr) b.par1
## b.pair1    -0.578
## s.mminutes  0.329 -0.333
## convergence code: 0
## singular fit
```

```
drop1(p_b.p_4, test = "Chisq")
```

Create PDF in your applications with the Pdfcrowd HTML to PDF API PDFCROWD

```
## singular fit
## singular fit
```

```
## Single term deletions
##
## Model:
## parasite ~ b.pair + s.mminutes + (1 | site:nestid) + (1 | observer)
##            Df    AIC    LRT Pr(Chi)
## <none>        240.94
## b.pair      1 244.59 5.6480 0.01747 *
## s.mminutes  1 242.87 3.9282 0.04748 *
## ---
## Signif. codes:  0 '***' 0.001 '**' 0.01 '*' 0.05 '.' 0.1 ' ' 1
```

```
test(lsmeans(p_b.p_4, ~ b.pair))
```

```
##  b.pair     lsmean       SE  df z.ratio p.value
##  0      -10.504271 1.342923 Inf  -7.822  <.0001
##  1       -7.755442 1.183869 Inf  -6.551  <.0001
##
## Results are given on the logit (not the response) scale.
```

```
Anova(p_b.p_4, type = "III")
```

```
## Error in Anova(p_b.p_4, type = "III"): could not find function "Anova"
```

```
vif.mer(p_b.p_4)
```

```
##    b.pair1 s.mminutes
##   1.124561   1.124561
```

Create PDF in your applications with the Pdfcrowd HTML to PDF API PDFCROWD

```
# plot

predicted.parasite <- predict(p_b.p_2, type = "response")

df.predicted.parasite <- data.frame(x = df$b.pair, y = predicted.parasite)

mean0 <- mean(df.predicted.parasite$y[df.predicted.parasite$x == 0])
mean1 <- mean(df.predicted.parasite$y[df.predicted.parasite$x == 1])

sd0 <- sd(df.predicted.parasite$y[df.predicted.parasite$x == 0])
sd1 <- sd(df.predicted.parasite$y[df.predicted.parasite$x == 1])

se0 <- sd0/sqrt(length(df[df$b.pair == 0,]))
se1 <- sd1/sqrt(length(df[df$b.pair == 1,]))

df.plot <- data.frame(matrix(c(mean0, mean1, 0, 1), nrow = 2, byrow = F, dimnames = list(c(1,2),c("mean", "alarm"
))))


means <- c(mean0, mean1)
names <- c("No alarm", "Alarm")
ses <- c(se0x, se1x)
plot.top <- max(means + ses*3)

par(mar = c(3,5,1,1))
pred.plot <- barplot(means, names.arg = names, col = c(r1, r3),  ylab = "Probability of parasitism (mean Â± SE)",
 ylim = c(0, 1), cex.lab=1.8, cex.axis=1.5, cex=1.8)

segments(pred.plot, means-ses, pred.plot, means+ses, lwd=1)

box()
```

Create PDF in your applications with the Pdfcrowd HTML to PDF API PDFCROWD

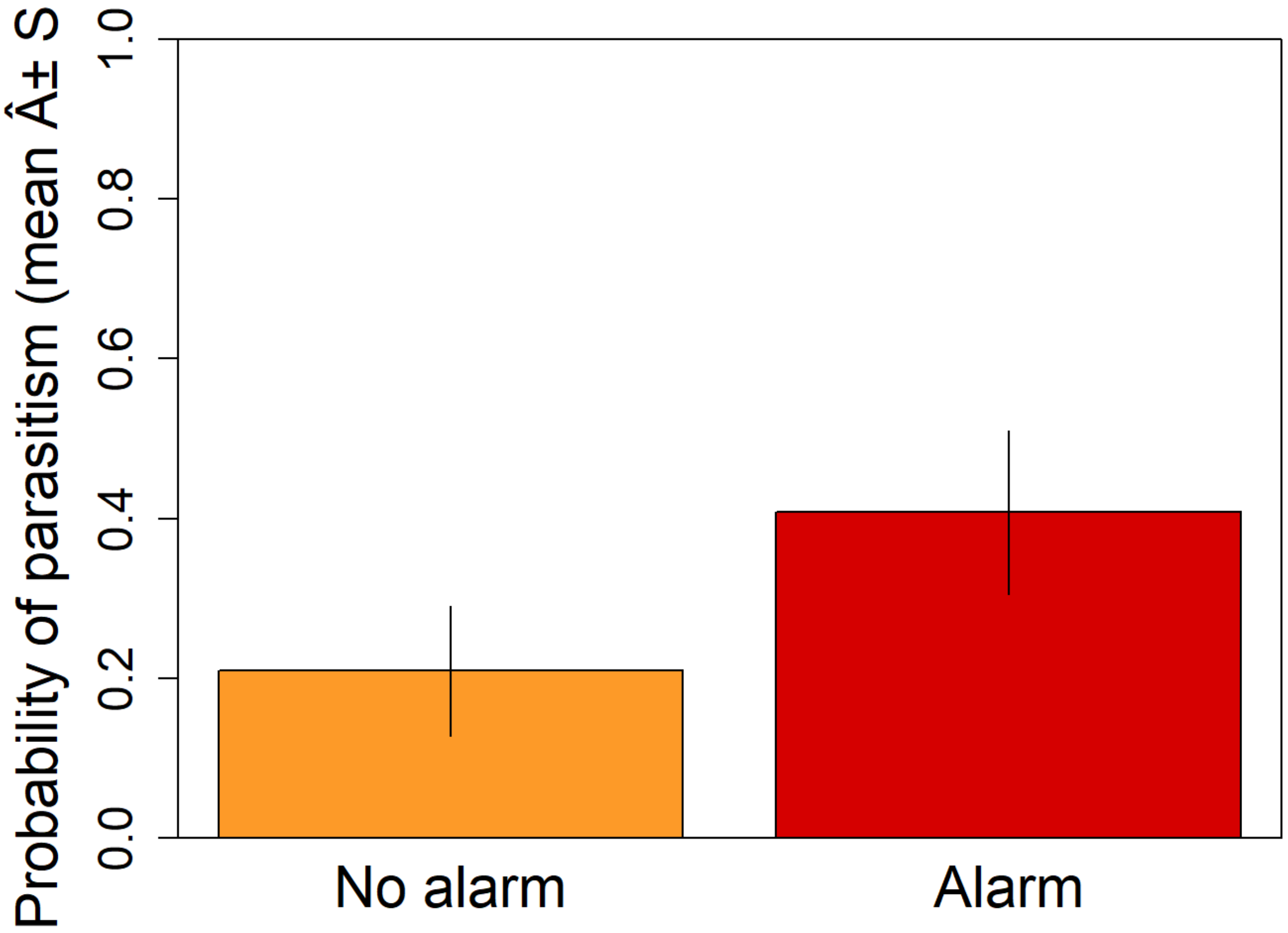


```
df2 <- read.csv("Experiment1-data.csv", header = T, row.names = 1, sep = ";")

df2$before.ccf <- as.numeric(as.character(df2$before.ccf))
df2$during.ccf <- as.numeric(as.character(df2$during.ccf))
df2$before.ccm <- as.numeric(as.character(df2$before.ccm))
df2$during.ccm <- as.numeric(as.character(df2$during.ccm))

table(df2$before.ccf)
```

Create PDF in your applications with the Pdfcrowd HTML to PDF API PDFCROWD

```
##
##  0  1  2
## 45  5  1
```

```
table(df2$during.ccf)
```

```
##
##  0  1  2  3
## 41  1  2  7
```

```
table(df2$before.ccm)
```

```
##
##  0  1  2  3
## 38  9  3  1
```

```
table(df2$during.ccm)
```

```
##
##  0  1  2  3
## 26  8  4 13
```

```
# common cuckoo males

df2$response.ccm <- df2$before.ccm + df2$during.ccm
df.plm <- df2[df2$response.ccm > "0",]

table(df.plm$before.ccm)
```

```
##
##  0  1  2  3
```

Create PDF in your applications with the Pdfcrowd HTML to PDF API PDFCROWD

```
## 17   9   3   1
```

```
table(df.plm$during.ccm)
```

```
##
##   0   1   2   3
##   5   8   4  13
```

```
male.matrix <- matrix(c(17,5,9,8,3,4,1,13), nrow = 4, byrow = T, dimnames = list(c("0","1","2","3"), c("before",
"during")))

fisher.test(t(male.matrix), alternative = "less")
```

```
##
##  Fisher's Exact Test for Count Data
##
## data:  t(male.matrix)
## p-value = 0.0002865
## alternative hypothesis: less
```

```
# common cuckoo females

df2$response.ccf <- df2$before.ccf + df2$during.ccf
df.plf <- df2[df2$response.ccf > "0",]

table(df.plf$before.ccf)
```

```
##
## 0 1 2
## 8 5 1
```

```
table(df.plf$during.ccf)
```

Create PDF in your applications with the Pdfcrowd HTML to PDF API PDFCROWD

```
##
## 0 1 2 3
## 4 1 2 7
```

```
female.matrix <-matrix(c(8,4,5,1,1,2,0,7), nrow = 4, byrow = T, dimnames = list(c("0","1","2","3"), c("before",
"during")))

fisher.test(t(female.matrix), alternative = "less")
```

```
##
##  Fisher's Exact Test for Count Data
##
## data:  t(female.matrix)
## p-value = 0.004043
## alternative hypothesis: less
```

```
# plot for Experiment 1

par(mar = c(1,1,1,1))
par(mfrow = c(2,1))
mosaicplot(female.matrix, main = "Female (n = 14)", sort = 2:1, cex.axis=1.5, col = c(r0, r1, r2, r3))
mosaicplot(male.matrix, main = "Male (n = 30)", sort = 2:1, cex.axis = 1.4, col = c(r0, r1, r2, r3),  xlab = "Res
ponse intensity")
```

Create PDF in your applications with the Pdfcrowd HTML to PDF API PDFCROWD

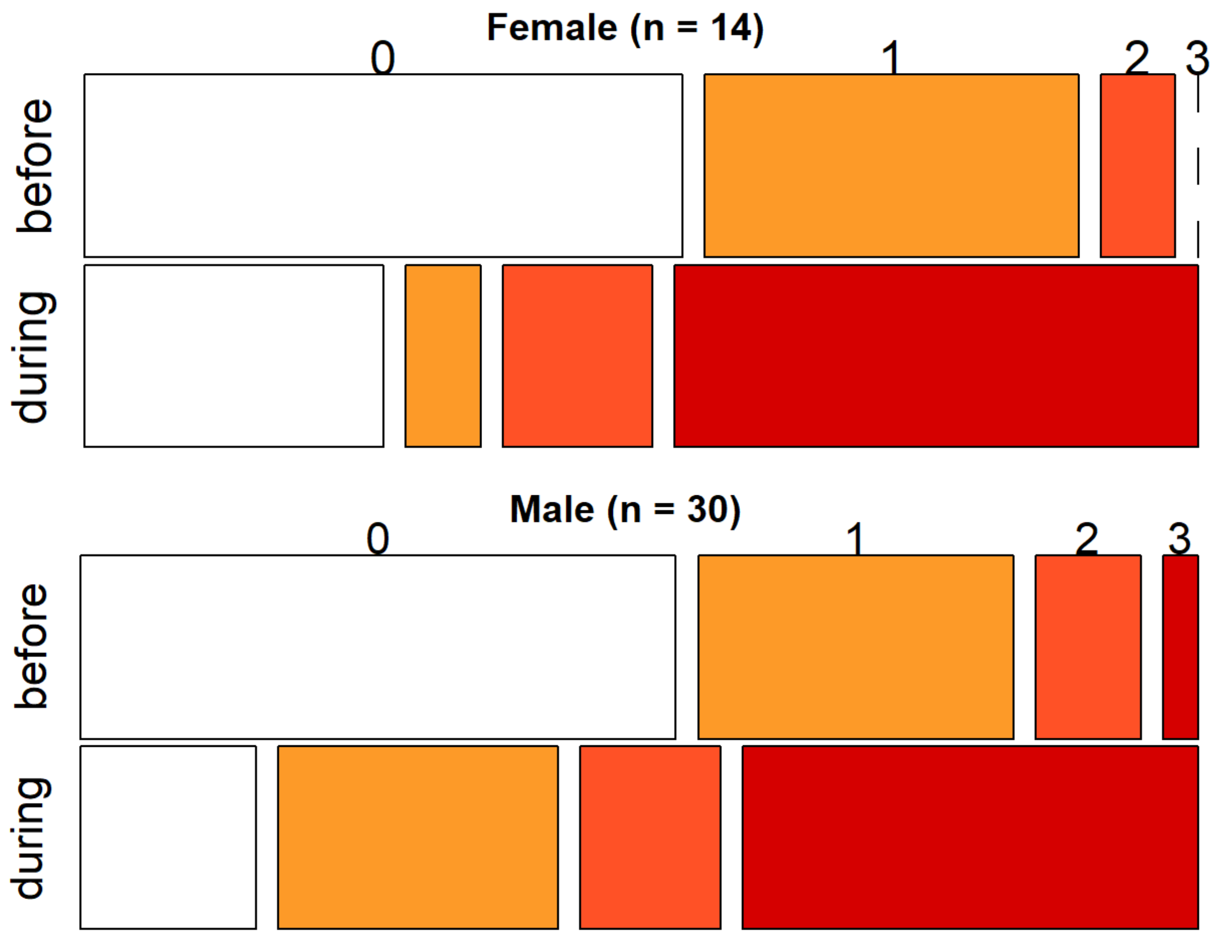


```
# common cuckoo females

female.matrix2 <- matrix(c(0, 5, 16, 11), nrow = 2, byrow = T, dimnames = list(c("positive","neutral"), c("contro
l", "experiment")))

fisher.test(female.matrix2, alternative = "less")
```

```
##
##  Fisher's Exact Test for Count Data
```

Create PDF in your applications with the Pdfcrowd HTML to PDF API PDFCROWD

```
##
## data:  female.matrix2
## p-value = 0.02169
## alternative hypothesis: true odds ratio is less than 1
## 95 percent confidence interval:
##  0.0000000 0.6958781
## sample estimates:
## odds ratio
##          0
```

```
# common cuckoo female, when one weaker response was considered as neutral
female.matrix2b <- matrix(c(0, 4, 16, 11), nrow = 2, byrow = T, dimnames = list(c("positive","neutral"), c("contr
ol", "experiment")))

fisher.test(female.matrix2b, alternative = "less")
```

```
##
##  Fisher's Exact Test for Count Data
##
## data:  female.matrix2b
## p-value = 0.04338
## alternative hypothesis: true odds ratio is less than 1
## 95 percent confidence interval:
##  0.0000000 0.9325457
## sample estimates:
## odds ratio
##          0
```

```
# common cuckoo males

male.matrix2 <- matrix(c(1, 7, 15, 9), nrow = 2, byrow = T, dimnames = list(c("positive","neutral"), c("control",
 "experiment")))

fisher.test(male.matrix2, alternative = "less")
```

Create PDF in your applications with the Pdfcrowd HTML to PDF API PDFCROWD

```
##
##  Fisher's Exact Test for Count Data
##
## data:  male.matrix2
## p-value = 0.01863
## alternative hypothesis: true odds ratio is less than 1
## 95 percent confidence interval:
##  0.0000000 0.7033189
## sample estimates:
## odds ratio
## 0.09243924
```

```
# plot for Experiment 2

par(mar = c(1,1,1,1))
par(mfrow = c(1,2))

mosaicplot(female.matrix2, col = c(r3, r1), main = "Female (n = 32)", sort = 2:1, cex.axis = 1.8, off = 5)
mosaicplot(male.matrix2, col = c(r3, r1), main = "Male (n = 32)", sort = 2:1, cex.axis = 1.8, off = 5)
```

Create PDF in your applications with the Pdfcrowd HTML to PDF API PDFCROWD

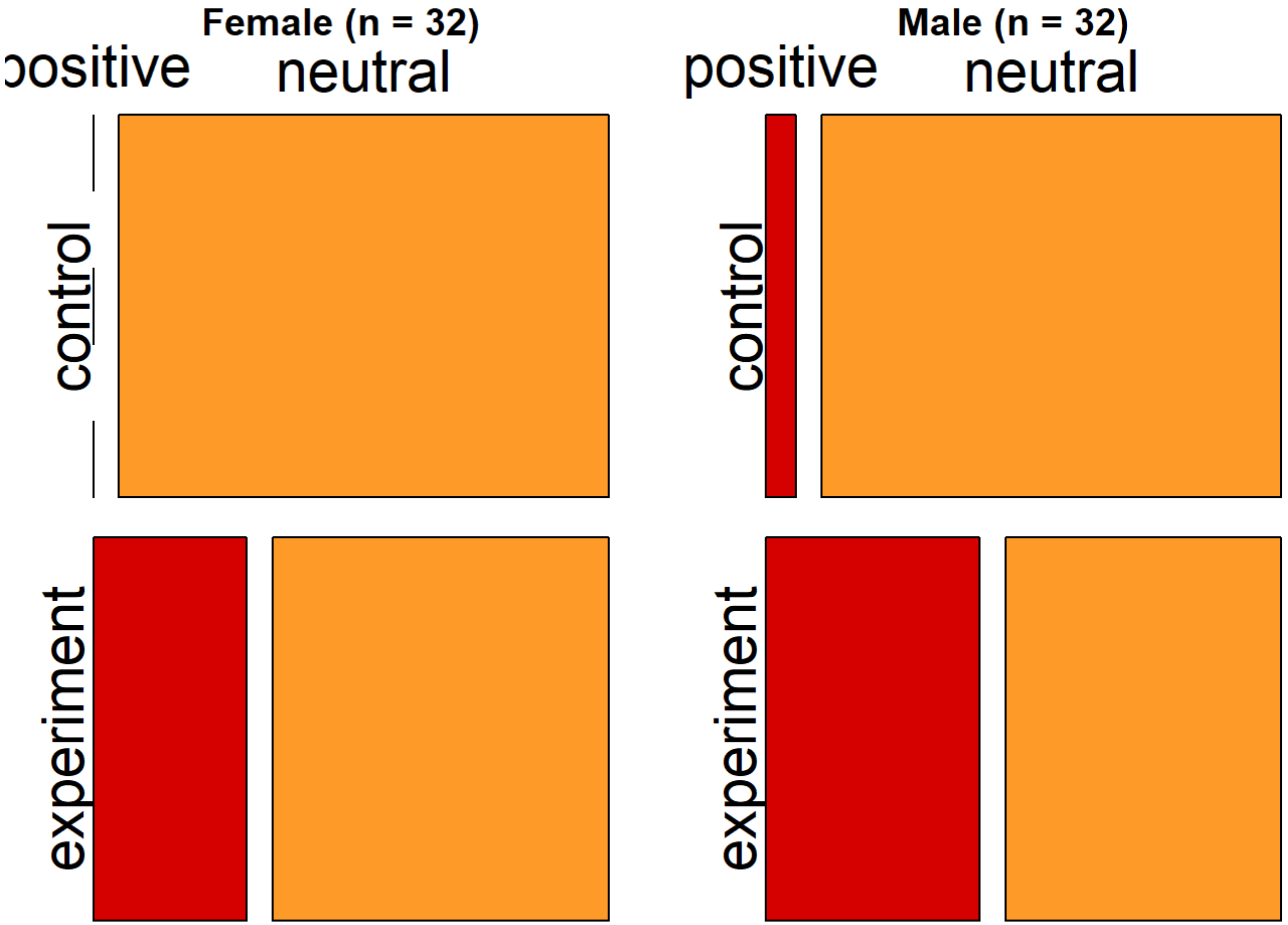


```
df3 <- read.csv("Drone-study-data.csv", header = T, row.names = 1, sep = ";")

# nest height
mean(df3$nest.height[df3$parasite == 0])
```

```
## [1] 13.75
```

```
sd(df3$nest.height[df3$parasite == 0])
```

Create PDF in your applications with the Pdfcrowd HTML to PDF API PDFCROWD

```
## [1] 4.49603
```

```
(sd(df3$nest.height[df3$parasite == 0])
)/mean(df3$nest.height[df3$parasite == 0])
```

```
## [1] 0.326984
```

```
median(df3$nest.height[df3$parasite == 0])
```

```
## [1] 12.5
```

```
c(min(df3$nest.height[df3$parasite == 0]), max(df3$nest.height[df3$parasite == 0]))
```

```
## [1] 10 24
```

```
mean(df3$nest.height[df3$parasite == 1])
```

```
## [1] 14.375
```

```
sd(df3$nest.height[df3$parasite == 1])
```

```
## [1] 2.615203
```

```
(sd(df3$nest.height[df3$parasite == 1])
)/mean(df3$nest.height[df3$parasite == 1])
```

```
## [1] 0.1819272
```

Create PDF in your applications with the Pdfcrowd HTML to PDF API PDFCROWD

```r
median(df3$nest.height[df3$parasite == 1])
```

```
## [1] 14
```

```r
c(min(df3$nest.height[df3$parasite == 1]),
max(df3$nest.height[df3$parasite == 1]))
```

```
## [1] 12 20
```

```r
wilcox.exact.default(df3$nest.height[df3$parasite == 0], df3$nest.height[df3$parasite == 1], alternative = "less"
)
```

```
##
##  Exact Wilcoxon rank sum test
##
## data:  df3$nest.height[df3$parasite == 0] and df3$nest.height[df3$parasite == 1]
## W = 22.5, p-value = 0.1719
## alternative hypothesis: true mu is less than 0
```

```r
# nest width
mean(df3$nest.width[df3$parasite == 0])
```

```
## [1] 10.375
```

```r
sd(df3$nest.width[df3$parasite == 0])
```

```
## [1] 0.5175492
```

Create PDF in your applications with the Pdfcrowd HTML to PDF API PDFCROWD

```
(sd(df3$nest.width[df3$parasite == 0])
)/mean(df3$nest.width[df3$parasite == 0])
```

```
## [1] 0.04988426
```

```
median(df3$nest.width[df3$parasite == 0])
```

```
## [1] 10
```

```
c(min(df3$nest.width[df3$parasite == 0]), max(df3$nest.width[df3$parasite == 0]))
```

```
## [1] 10 11
```

```
mean(df3$nest.width[df3$parasite == 1])
```

```
## [1] 10.375
```

```
sd(df3$nest.width[df3$parasite == 1])
```

```
## [1] 1.06066
```

```
(sd(df3$nest.width[df3$parasite == 1])
)/mean(df3$nest.width[df3$parasite == 1])
```

```
## [1] 0.1022323
```

```
median(df3$nest.width[df3$parasite == 1])
```

Create PDF in your applications with the Pdfcrowd HTML to PDF API PDFCROWD

```
## [1] 10.5
```

```
c(min(df3$nest.width[df3$parasite == 1]),
max(df3$nest.width[df3$parasite == 1]))
```

```
## [1]  9 12
```

```
wilcox.exact.default(df3$nest.width[df3$parasite == 0], df3$nest.width[df3$parasite == 1], alternative = "less")
```

```
##
##  Exact Wilcoxon rank sum test
##
## data:  df3$nest.width[df3$parasite == 0] and df3$nest.width[df3$parasite == 1]
## W = 31.5, p-value = 0.5
## alternative hypothesis: true mu is less than 0
```

```
# nest volume

df3$nest.volume <- 3.14 * df3$nest.height * ((df3$nest.width/2)^2)
df3$nest.volume
```

```
##  [1]  785.000 1020.500 1899.700 1234.805 2279.640 1099.000  763.020
##  [8]  953.775 1695.600  942.000 1139.820  785.000  942.000 1424.775
## [15] 1424.775 1020.500
```

```
mean(df3$nest.volume[df3$parasite == 0])
```

```
## [1] 1184.467
```

```
sd(df3$nest.volume[df3$parasite == 0])
```

Create PDF in your applications with the Pdfcrowd HTML to PDF API PDFCROWD

```
## [1] 488.6239
```

```r
(sd(df3$nest.volume[df3$parasite == 0])
)/mean(df3$nest.volume[df3$parasite == 0])
```

```
## [1] 0.4125264
```

```r
median(df3$nest.volume[df3$parasite == 0])
```

```
## [1] 1059.75
```

```r
c(min(df3$nest.volume[df3$parasite == 0]), max(df3$nest.volume[df3$parasite == 0]))
```

```
## [1]  785.00 2279.64
```

```r
mean(df3$nest.volume[df3$parasite == 1])
```

```
## [1] 1241.772
```

```r
sd(df3$nest.volume[df3$parasite == 1])
```

```
## [1] 400.5956
```

```r
(sd(df3$nest.volume[df3$parasite == 1])
)/mean(df3$nest.volume[df3$parasite == 1])
```

```
## [1] 0.3226
```

Create PDF in your applications with the Pdfcrowd HTML to PDF API PDFCROWD

```
median(df3$nest.volume[df3$parasite == 1])
```

```
## [1] 1127.653
```

```
c(min(df3$nest.volume[df3$parasite == 1]),
max(df3$nest.volume[df3$parasite == 1]))
```

```
## [1]  763.02 1899.70
```

```
wilcox.exact.default(df3$nest.volume[df3$parasite == 0], df3$nest.volume[df3$parasite == 1], alternative = "less"
)
```

```
##
##  Exact Wilcoxon rank sum test
##
## data:  df3$nest.volume[df3$parasite == 0] and df3$nest.volume[df3$parasite == 1]
## W = 28.5, p-value = 0.3706
## alternative hypothesis: true mu is less than 0
```

```
# nest distance from water
mean(df3$dist.water[df3$parasite == 0])
```

```
## [1] 70
```

```
sd(df3$dist.water[df3$parasite == 0])
```

```
## [1] 20.35401
```

Create PDF in your applications with the Pdfcrowd HTML to PDF API PDFCROWD

```
sd(df3$dist.water[df3$parasite == 0])/
mean(df3$dist.water[df3$parasite == 0])
```

```
## [1] 0.2907716
```

```
median(df3$dist.water[df3$parasite == 0])
```

```
## [1] 62.5
```

```
c(min(df3$dist.water[df3$parasite == 0]), max(df3$dist.water[df3$parasite == 0]))
```

```
## [1]  50 110
```

```
mean(df3$dist.water[df3$parasite == 1])
```

```
## [1] 78.125
```

```
sd(df3$dist.water[df3$parasite == 1])
```

```
## [1] 26.98379
```

```
sd(df3$dist.water[df3$parasite == 1])/
mean(df3$dist.water[df3$parasite == 1])
```

```
## [1] 0.3453925
```

```
median(df3$dist.water[df3$parasite == 1])
```

Create PDF in your applications with the Pdfcrowd HTML to PDF API PDFCROWD

```
## [1] 80
```

```
c(min(df3$dist.water[df3$parasite == 1]), max(df3$dist.water[df3$parasite == 1]))
```

```
## [1]  40 115
```

```
wilcox.exact.default(df3$dist.water[df3$parasite == 0], df3$dist.water[df3$parasite == 1], alternative = "greate
r")
```

```
##
##  Exact Wilcoxon rank sum test
##
## data:  df3$dist.water[df3$parasite == 0] and df3$dist.water[df3$parasite == 1]
## W = 26, p-value = 0.7399
## alternative hypothesis: true mu is greater than 0
```

```
# distance from bank
mean(df3$dist.bank[df3$parasite == 0])
```

```
## [1] 238.75
```

```
sd(df3$dist.bank[df3$parasite == 0])
```

```
## [1] 72.19765
```

```
sd(df3$dist.bank[df3$parasite == 0])/
mean(df3$dist.bank[df3$parasite == 0])
```

```
## [1] 0.3023985
```

Create PDF in your applications with the Pdfcrowd HTML to PDF API PDFCROWD

```
median(df3$dist.bank[df3$parasite == 0])
```

```
## [1] 245
```

```
c(min(df3$dist.bank[df3$parasite == 0]), max(df3$dist.bank[df3$parasite == 0]))
```

```
## [1] 100 320
```

```
mean(df3$dist.bank[df3$parasite == 1])
```

```
## [1] 178.125
```

```
sd(df3$dist.bank[df3$parasite == 1])
```

```
## [1] 39.27354
```

```
sd(df3$dist.bank[df3$parasite == 1])/
mean(df3$dist.bank[df3$parasite == 1])
```

```
## [1] 0.220483
```

```
median(df3$dist.bank[df3$parasite == 1])
```

```
## [1] 170
```

```
c(min(df3$dist.bank[df3$parasite == 1]), max(df3$dist.bank[df3$parasite == 1]))
```

Create PDF in your applications with the Pdfcrowd HTML to PDF API PDFCROWD

```
## [1] 125 240
```

```
wilcox.exact.default(df3$dist.bank[df3$parasite == 0], df3$dist.bank[df3$parasite == 1], alternative = "greater")
```

```
##
##  Exact Wilcoxon rank sum test
##
## data:  df3$dist.bank[df3$parasite == 0] and df3$dist.bank[df3$parasite == 1]
## W = 50.5, p-value = 0.02525
## alternative hypothesis: true mu is greater than 0
```

```
# nest height above water
mean(df3$above.water[df3$parasite == 0])
```

```
## [1] 82.875
```

```
sd(df3$above.water[df3$parasite == 0])
```

```
## [1] 29.12259
```

```
sd(df3$above.water[df3$parasite == 0])/
mean(df3$above.water[df3$parasite == 0])
```

```
## [1] 0.3514037
```

```
median(df3$above.water[df3$parasite == 0])
```

```
## [1] 83.5
```

Create PDF in your applications with the Pdfcrowd HTML to PDF API PDFCROWD

```
c(min(df3$above.water[df3$parasite == 0]), max(df3$above.water[df3$parasite == 0]))
```

```
## [1]  40 120
```

```
mean(df3$above.water[df3$parasite == 1])
```

```
## [1] 79.625
```

```
sd(df3$above.water[df3$parasite == 1])
```

```
## [1] 25.76231
```

```
sd(df3$above.water[df3$parasite == 1])/
mean(df3$above.water[df3$parasite == 1])
```

```
## [1] 0.3235454
```

```
median(df3$above.water[df3$parasite == 1])
```

```
## [1] 82.5
```

```
c(min(df3$above.water[df3$parasite == 1]), max(df3$above.water[df3$parasite == 1]))
```

```
## [1]  42 118
```

```
wilcox.exact.default(df3$above.water[df3$parasite == 0], df3$above.water[df3$parasite == 1], alternative = "less"
)
```

Create PDF in your applications with the Pdfcrowd HTML to PDF API PDFCROWD

```
##
##  Exact Wilcoxon rank sum test
##
## data:  df3$above.water[df3$parasite == 0] and df3$above.water[df3$parasite == 1]
## W = 33.5, p-value = 0.5712
## alternative hypothesis: true mu is less than 0
```

```
# vegetation height above water
mean(df3$above.nest[df3$parasite == 0])
```

```
## [1] 178.75
```

```
sd(df3$above.nest[df3$parasite == 0])
```

```
## [1] 25.31939
```

```
sd(df3$above.nest[df3$parasite == 0])/
mean(df3$above.nest[df3$parasite == 0])
```

```
## [1] 0.1416469
```

```
median(df3$above.nest[df3$parasite == 0])
```

```
## [1] 190
```

```
c(min(df3$above.nest[df3$parasite == 0]), max(df3$above.nest[df3$parasite == 0]))
```

```
## [1] 140 200
```

Create PDF in your applications with the Pdfcrowd HTML to PDF API PDFCROWD

```
mean(df3$above.nest[df3$parasite == 1])
```

```
## [1] 200
```

```
sd(df3$above.nest[df3$parasite == 1])
```

```
## [1] 45.66962
```

```
sd(df3$above.nest[df3$parasite == 1])/
mean(df3$above.nest[df3$parasite == 1])
```

```
## [1] 0.2283481
```

```
median(df3$above.nest[df3$parasite == 1])
```

```
## [1] 200
```

```
c(min(df3$above.nest[df3$parasite == 1]), max(df3$above.nest[df3$parasite == 1]))
```

```
## [1] 140 280
```

```
wilcox.exact.default(df3$above.nest[df3$parasite == 0], df3$above.nest[df3$parasite == 1], alternative = "greate
r")
```

```
##
##  Exact Wilcoxon rank sum test
##
## data:  df3$above.nest[df3$parasite == 0] and df3$above.nest[df3$parasite == 1]
```

Create PDF in your applications with the Pdfcrowd HTML to PDF API PDFCROWD

```
## W = 22.5, p-value = 0.8532
## alternative hypothesis: true mu is greater than 0
```

```
# distance from closest perch
mean(df3$perch.dist[df3$parasite == 0])
```

```
## [1] 11.75
```

```
sd(df3$perch.dist[df3$parasite == 0])
```

```
## [1] 5.574175
```

```
sd(df3$perch.dist[df3$parasite == 0])/
mean(df3$perch.dist[df3$parasite == 0])
```

```
## [1] 0.4743979
```

```
median(df3$perch.dist[df3$parasite == 0])
```

```
## [1] 12
```

```
c(min(df3$perch.dist[df3$parasite == 0]), max(df3$perch.dist[df3$parasite == 0]))
```

```
## [1]  3 20
```

```
mean(df3$perch.dist[df3$parasite == 1])
```

```
## [1] 10.75
```

Create PDF in your applications with the Pdfcrowd HTML to PDF API PDFCROWD

```r
sd(df3$perch.dist[df3$parasite == 1])
```

```
## [1] 6.923046
```

```r
sd(df3$perch.dist[df3$parasite == 1])/
mean(df3$perch.dist[df3$parasite == 1])
```

```
## [1] 0.6440043
```

```r
median(df3$perch.dist[df3$parasite == 1])
```

```
## [1] 8
```

```r
c(min(df3$perch.dist[df3$parasite == 1]), max(df3$perch.dist[df3$parasite == 1]))
```

```
## [1]  5 25
```

```r
wilcox.exact.default(df3$perch.dist[df3$parasite == 0], df3$perch.dist[df3$parasite == 1], alternative = "less")
```

```
##
##  Exact Wilcoxon rank sum test
##
## data:  df3$perch.dist[df3$parasite == 0] and df3$perch.dist[df3$parasite == 1]
## W = 36.5, p-value = 0.6886
## alternative hypothesis: true mu is less than 0
```

```r
#nest visibility
nv <- t(matrix(c(4,5,3,3,1,0), nrow = 3, byrow = T, dimnames = list( c("direct nest view", "indirect nest view",
```

Create PDF in your applications with the Pdfcrowd HTML to PDF API PDFCROWD

```r
"no nest view"), c("unparasited","parasited"))))
fisher.test(nv, alternative = "less")
```

```
##
##  Fisher's Exact Test for Count Data
##
## data:  nv
## p-value = 1
## alternative hypothesis: less
```

Create PDF in your applications with the Pdfcrowd HTML to PDF API PDFCROWD
